# The correlation between the change of Hounsfield units value and Modic changes in the lumbar vertebral endplate

**DOI:** 10.1186/s12891-021-04330-5

**Published:** 2021-06-02

**Authors:** Jiandong Zhu, Hao Wu, Yilei Chen, Junhui Liu, Zhi Shan, Shunwu Fan, Fengdong Zhao

**Affiliations:** 1 Department of Orthopaedic Surgery, Sir Run Run Shaw Hospital, Zhejiang University School of Medicine, No. 3, Qingchun Rd East, Hangzhou, 310016 China; 2 Tongxiang first people’s Hospital, Tongxiang, China; 3 Key Laboratory of Musculoskeletal System Degeneration and Regeneration Translational Research of Zhejiang Province, Hangzhou, China; 4 Department of Orthopaedics and Traumatology, The University of Hong Kong, Pokfulam, Hong Kong, SAR, China

**Keywords:** Modic changes, HU value, Area ratio, BMD, Disc degeneration

## Abstract

**Objectives:**

To evaluate the changes of Hounsfield units (HU) value in different types of Modic changes (MCs) and to analyze the correlation between the change of HU value and area ratio of MCs region, bone mineral density (BMD), and degree of intervertebral disc degeneration.

**Methods:**

One hundred fifty-eight endplates with MCs were included and analyzed. HU values of MCs regions and adjacent vertebral corresponding regions without MCs were measured. The area ratio of MCs region was defined as the area of MCs divided by the area of endplate or the vertebral sagittal plane. BMD was measured by Dual-energy x-ray absorptiometry (DXA). Degree of intervertebral disc degeneration was evaluated based on Pfirrmann classification. According to the types of variables, descriptive statistics, Kolmogorove-Smirnov test, paired t-test, Wilcoxon signed-rank test, Independent-Samples T Test, and Pearson correlation analysis were used.

**Results:**

The HU values in any types of MCs are significantly higher than that of adjacent vertebral corresponding regions without MCs (*P* < 0.001). The HU value of the type III MCs is higher than that of the type I and type II MCs. HU value was positively correlated with BMD. In the levels with Grade V disc degeneration, the area ratio of MCs region was significant increased.

**Conclusions:**

HU values of the vertebral endplate and bone marrow were increased in most MCs regions with all types of MCs. HU value of endplates had a significantly positive correlation with BMD. Higher area ratio of MCs region is associated with more severe intervertebral disc degeneration.

## Key points

Measure the changes of Hounsfield units (HU) value in different types of Modic changes (MCs), evaluate endplate sclerosis;

The correlation between the change of HU value and area ratio of MCs region, bone mineral density (BMD), and degree of intervertebral disc degeneration.

Higher area ratio of MCs region, more severe intervertebral disc degeneration, more severe chronic lower back pain.

## Background

The Modic changes (MCs) of lumbar spine, first reported by de Roos et al. [1] in 1987, refer to the signal changes of the lumbar endplate and bone marrow under endplate on Magnetic Resonance Imaging (MRI) scans. Modic et al. described the types of signal changes, classification criteria and pathological changes systematically in l988 [2]. MCs were classified into three types: type I lesions present low T1 and high T2 signals and indicate an ongoing active degenerative process with vascularised fibrous tissue within the bone marrow, which are thought to indicate inflammatory reactions; type II lesions involve high T1 and T2 signals and reflect fatty replacement of the bone marrow, which are regarded as a stable and chronic fat deposition; type III lesions involve low T1 and T2 signals and are thought to be associated with endplate sclerosis [2, 3]. In recent years, due to the popularization of MRI in the diagnosis of lumbar degenerative diseases, more cases of MRI signals changes in endplate and bone marrow have been found. A lot of studies in current literature described the etiology, epidemiology and clinical relevance of MCs. However, there are few studies on the correlation between MCs and Hounsfield units value of vertebral marrow and endplate.

MRI scan can show different tissues via various scanning parameters and can be used for evaluation of MCs. Computed tomography (CT) provides images of the bony structure and precise HU value of tissues, which allows further evaluation of osteosclerosis in the endplate and bone marrow under the endplate with MCs. We hypothesized that besides signal changes in MRI, kinds of types MCs would show a different HU value in CT. Our study aimed to evaluate the HU values changes of all three types of MCs, to find their association with osteosclerosis in the endplate and bone marrow, and to analyze the correlation between the change of HU value and area ratio of MCs region, BMD, and degree of intervertebral disc degeneration.

## Methods

### Patients

Our study included 62 consecutive inpatients with lumbar degenerative disease, who were admitted to the Department of Orthopaedics at the author’s hospital between August 2018 and August 2019. All patients had preoperative MRI indicating MCs, and were treated with lumbar interbody fusion because of the failure of normal conservative treatment. All 62 patients (35 women and 27 men; mean age, 62.61 ± 11.44 years; age range,30–86 years; mean body mass index,24.03 ± 2.22;BMI range,19.05–31.11) underwent CT, MRI and Dual-energy x-ray absorptiometry (DXA) examinations, which were evaluated retrospectively. Patients were divided into different groups according to the comparison parameters. Patients with lumbar fractures, a history of lumbar surgery, spinal infection, severe spinal deformities, and mixed types of MCs were excluded.

The ethical approval was obtained from the medical ethics committee of the hospital. Every patient had a written informed consent for his information to be stored in the hospital’s database and used for study.

### Computed tomography, magnetic resonance imaging and dual-energy x-ray

The CT images of the lumbar spine were obtained using a 64-slice CT scanner (GE LightSpeed; GE Healthcare) with a detector configuration of 64 × 1.25 mm. A standard lumbar spine protocol with a tube voltage of 120 kV, tube current of 100–650 mA and rotation time of 0.8 s was used. Automatic tube current modulation based on the patient’s size and X-ray attenuation was used. The slice thickness and reconstruction interval were 1.25 mm and 0.625 mm, respectively.

MRI of the lumbar spine was performed at our hospital using a General Electric 1.5-T magnet with a T1-weighted sequence (repetition time/echo time, 560 ms/12 ms; field of view, 320 × 256; receiver bandwidth, variable; 4.0-mm slice with a gap of 1.0 mm; number of excitations, 3) and a T2-weighted sequence (repetition time/echo time,3000 ms/100 ms; field of view, 320 × 256; receiver bandwidth, variable; 4.0-mm slice with a gap of 1.0 mm; number of excitations, 3).

Dual-energy x-ray absorptiometry was performed using standard techniques on Lunar Prodigy densitometers (GE Healthcare, Waukesha, WI). Central DXA BMD T-scores were recorded from the lumbar spine and hip.

### Imaging evaluation

**Fig. 1:**
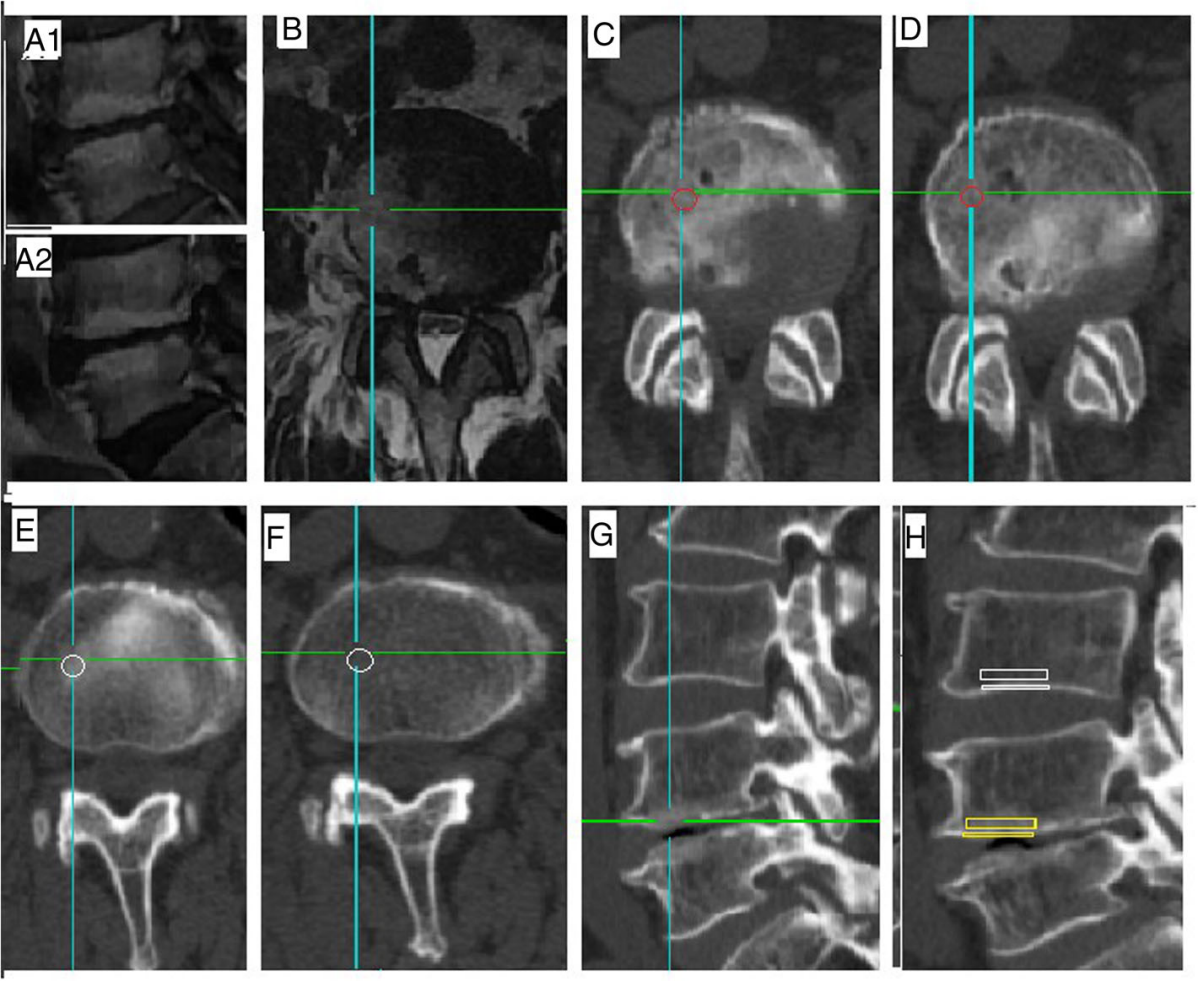
Measurement of HU value. A1/A2, signal changes of endplate and bone marrow on MRI scans (corresponding T1W/T2W); B, locate MCs regions in axial plane; C/D, oval ROI of HU value in axial plane with MCs (corresponding endplate/bone marrow); E/F, oval ROI of adjacent vertebra without MCs (corresponding endplate/bone marrow); G, located MCs regions in sagittal CT plane; H, rectangular ROI in sagittal CT plane (small yellow rectangle showed endplate with MCs, big yellow rectangle showed bone marrow with MCs; small white rectangle showed endplate with no MCs, big white rectangle showed bone marrow with no MCs)

**Fig. 2:**
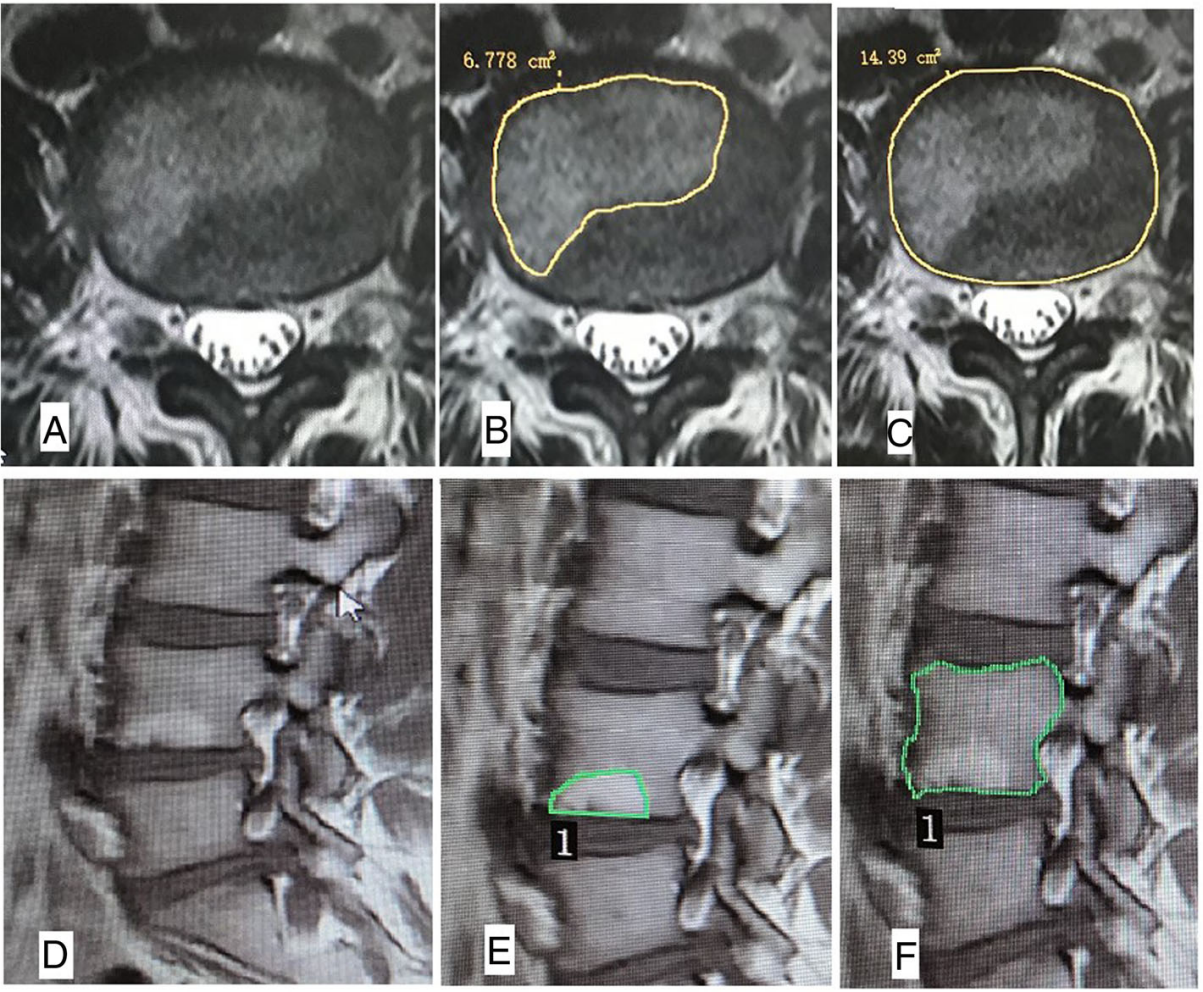
Measurement of the area ratio of MCs region. **a**, endplate with MCs; **b**, locating and measuring the area of MCs region; **c**, locating and measuring the area of the endplate; **d**, MCs in sagittal plane; **e**, locating and measuring the area of MCs in sagittal plane; **f**, locating and measuring the area of vertebral sagittal plane. The axial area ratio = B/C, the sagittal area ratio = E/F

Image evaluation was performed by an experienced radiologist and an orthopaedic surgeon who were blinded to the patient information. The evaluation included the presence, position, area ratio and classification of MCs using MRI, the HU value of endplate and bone marrow from CT scans. HU values of the bone marrow regions and endplate from MCs regions and corresponding regions in adjacent no-MCs vertebra in the sagittal plane and axial plane were measured in reconstructed CT images. BMD was measured by DXA. Degree of intervertebral disc degeneration was evaluated based on Pfirrmann classification from MRI T2WI [4]. The HU value measurement position was the endplate and the bone marrow. An oval region of interest (ROI) was used in the axial plane, and a rectangular region of interest (ROI) was used in the sagittal plane. The choice of sagittal and axial CT plane was based on the sagittal and axial MRI plane showing clear MCs. If an ideal MCs region was found in the sagittal and axial plane in MR, the position of this plane on the MRI plane was marked. We then identify the same position on the CT images in the sagittal and axial CT plane that contained this MCs. A region of interest (ROI) was marked on sagittal and axial CT planes within the MCs regions and corresponding regions in adjacent no-MCs vertebra (Fig. 1). The mean HU value of the ROI was calculated. The axial area ratio of MCs region was defined as the maximum area of MCs in the axial plane of the endplate divided by the area of endplate. The sagittal area ratio was defined as the maximum area of MCs in the sagittal plane divided by the area of the vertebral sagittal plane from MRI. We measured the area ratio of MCs region based on MRI scans of the axial plane and sagittal plane (Fig. 2). BMD assessment standard: osteopenia (T-score between − 1.0 and − 2.5 SD) or osteoporosis (T-score ≤ − 2.5 SD) was based on the lowest T-score from either femoral or vertebral region [5, 6]. Pfirrmann classification of disc degeneration was divided into Grade I to Grade V [4]. All the HU values, the area ratios of MCs region, T-score and degree of disc degeneration were measured twice by an experienced radiologist and orthopaedic surgeon (kappa> 0.8). According to these data, we calculated the average value, which was used in the statistical analysis.

### Statistical analysis

All patient’s information, CT and MRI image data, the axial and sagittal area ratios of MCs region, T-score and degree of disc degeneration were collected in an Excel database (Microsoft Corp.) and analyzed statistically using SPSS 18.0 software (PASW Statistics, IBM Corp.). Descriptive statistics were used to describe the age, gender, BMI of the patients, the number and distribution of MCs types. Continuous variables are presented as the mean ± standard deviation (SD). The HU values of the vertebral endplate and bone marrow showed a normal distribution with in each group. Kolmogorove-Smirnov test was used to assess interobserver reliability. The differences of HU values between MCs regions and corresponding regions with no MCs in adjacent vertebra were determined using the paired t-test and Wilcoxon signed-rank test. Differences in HU value and the area ratio of MCs region among kinds type of MCs and degree of disc degeneration were compared using an independent-Samples T Test. Correlation of the change of HU value, the area ratio of MCs region, BMD and degree of disc degeneration were compared using Pearson correlation analysis. A *p* value < 0.05 was considered statistically significant.

## Results

### Demography of patient and evaluation of Modic changes

**Fig. 3:**
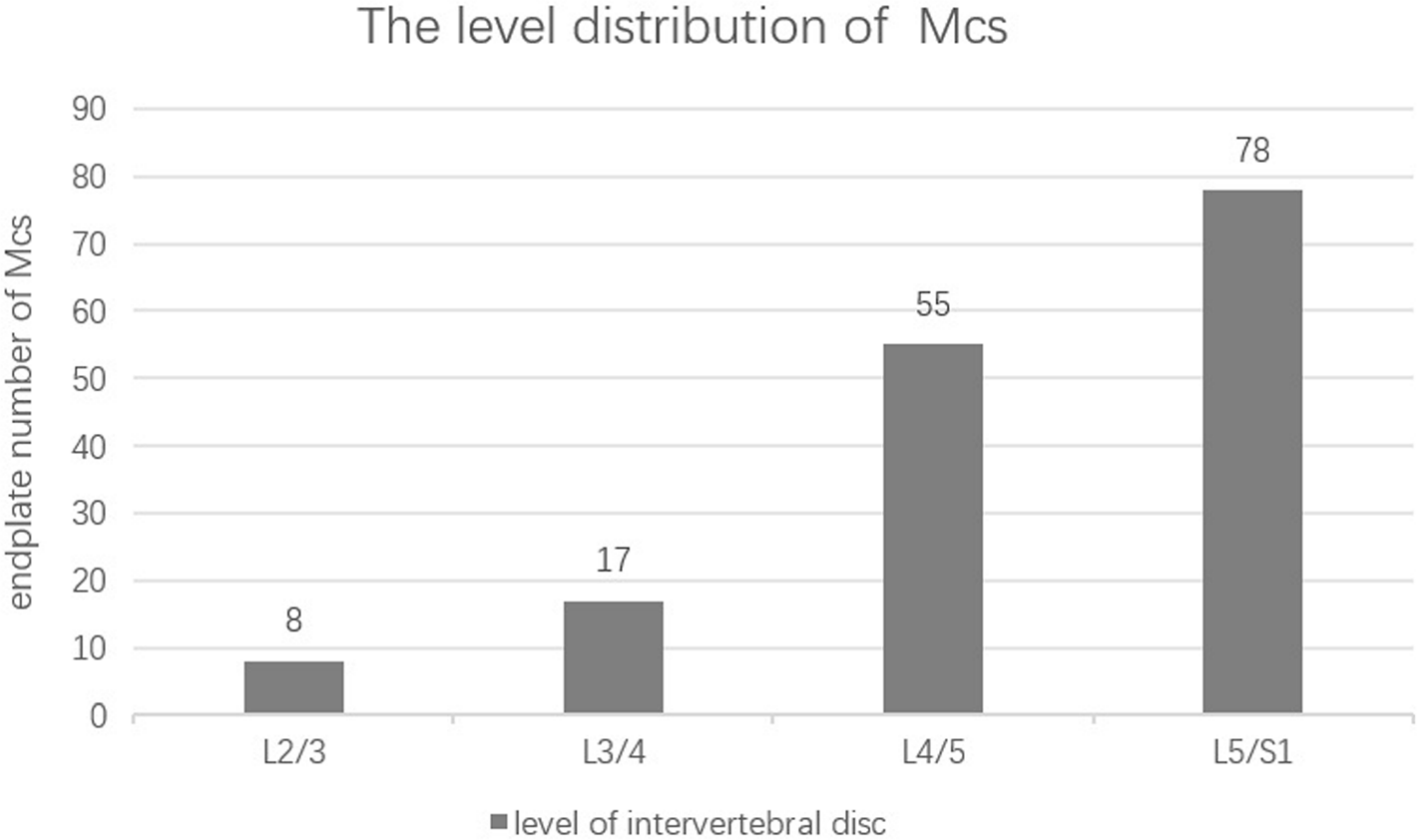
The level distribution of MCs. MCs mostly occur at the L5-S1 level (78/158, 49.4%) and L4/5 level (55/158, 34.8%)

Sixty-two patients were included in this study (Table 1). All patients had MCs at one or more endplate levels. Of 62 patients, MCs exists in 158 endplates. Type I, II and III MCs were seen in 21 (13.3%), 125 (79.1%) and12 (7.6%) endplates, respectively. MCs mostly occurs in the L5-S1 level (78/158, 49.4%) andL4/5 level (55/158, 34.8%) (Fig. 3). The distribution of MCs in our study was in accordance with previous studies [7]. Patients with type I, II and III MCs were no statistical difference in age, gender, BMI.

**Table 1:** Demography of patients

Demography of Patients	
Sample Size of Patients	62
Number of MCs endplates	158
Gender (Male/Female)	27/35
Age	62.61 ± 11.44 (30–86)
BMI	24.03 ± 2.22 (19.05–31.11)

### Evaluation of HU values and BMD

HU values of MCs regions and corresponding regions in adjacent vertebra with no-MCs are displayed in Table 2. We compared different Modic types and found that the HU values of MCs regions were significantly higher than that of corresponding regions in adjacent vertebra with no-MCs for both endplate and bone marrow. In the axial and sagittal plane, we got similar results. The details were as follows: 1) type I, endplates regions (axial plane: 543.70 ± 130.02HU versus 351.88 ± 90.72HU, *P* < 0.001, sagittal plane: 546.95 ± 123.50HU versus 350.27 ± 87.37HU, *P* < 0.001) and bone marrow regions (axial plane: 339.32 ± 139.82HU versus 147.28 ± 36.52HU, *P* < 0.001, sagittal plane: 332.51 ± 144.24HU versus 142.77 ± 41.23HU, *P* < 0.001); 2) type II, endplates regions (axial plane: 561.90 ± 158.40HU versus 327.66 ± 87.32HU, *P* < 0.001, sagittal plane: 565.30 ± 155.27HU versus 326.17 ± 85.39HU, *P* < 0.001) and bone marrow regions (axial plane: 354.05 ± 148.54HU versus 144.11 ± 52.68HU, *P* < 0.001, sagittal plane: 357.75 ± 149.05HU versus 147.72 ± 52.19HU, P < 0.001); 3) type III, endplates regions (axial plane: 729.87 ± 125.83HU versus 392.28 ± 107.28HU, *P* < 0.001, sagittal plane: 736.93 ± 139.58 HU versus 391.71 ± 99.01HU, *P* < 0.001) and bone marrow regions (axial plane: 532.90 ± 186.24 HU versus 145.08 ± 41.88HU, *P* < 0.001, sagittal plane: 544.32 ± 192.60HU versus 143.28 ± 41.13HU, *P* < 0.001). Furthermore, the HU of type III MCs is higher than that of type I, II MCs (*P* < 0.01), while the HU in type I and II MCs showed no significant difference (*P* > 0.05).

**Table 2:** HU values of MC regions and adjacent no MC vertebra corresponding regions

HU value (HU)	Endplate regions		Bone marrow regions	
	axial plane	sagittal plane	axial plane	sagittal plane
MCs I	543.70 ± 130.02	546.95 ± 123.50	339.32 ± 139.82	332.51 ± 144.24
Adjacent no MCs I	351.88 ± 90.72	350.27 ± 87.37	147.28 ± 36.52	142.77 ± 41.23
MCs II	561.90 ± 158.40	565.30 ± 155.27	354.05 ± 148.54	357.75 ± 149.05
Adjacent no MCs II	327.66 ± 87.32	326.17 ± 85.39	144.11 ± 52.68	147.72 ± 52.19
MCs III	729.87 ± 125.83	736.93 ± 139.58	532.90 ± 186.24	544.32 ± 192.60
Adjacent no MCs III	392.28 ± 107.28	391.71 ± 99.01	145.08 ± 41.88	143.28 ± 41.13

The HU values of MC regions and adjacent no MC vertebra corresponding regions were measured in different adjacent vertebral levels in the same patient at the same time and on the same CT image

We compared BMD with CT value of lumbar spine. Based on the lowest T-score in their vertebral region, subjects were divided into three groups: osteopenia (T-score between − 1.0 and − 2.5 SD) or osteoporosis (T-score ≤ − 2.5 SD) or normal (T-score ≥ − 1.0 SD). Our study showed that the HU value of endplate was significant positively correlated with BMD (r = 0.467, *p* < 0.001), and the mean HU value of endplate was significantly different in three groups (*p* < 0.001). The mean HU value was highest in normal group, and lowest in osteoporosis group. Specific data was shown in Table 3.

**Table 3:** The mean HU value of BMD groups

BMD group	Osteoporosis group	Osteopenia group	Normal group
Mean T-score	−2.70 ± 0.20	−1.46 ± 0.37	−0.38 ± 0.60
Mean HU value	401.46 ± 104.46**	545.70 ± 123.52**	659.86 ± 156.17**

**The HU value of endplate was significant positively correlated with T-score (*r* = 0.467, *p* < 0.001), and the mean HU value of endplate was significantly different in three groups (*p* < 0.001)

### Evaluation of the area ratio of Modic change region

Since the size of each patient’s vertebral body is different, we use area ratio to assess the areas of MCs with more accuracy. The area ratio of MCs region was measured in the axial plane of the endplate and in the sagittal plane of the vertebral body from MRI scans. The area ratio information of MCs for all 158 endplates were displayed in Table 4. We evaluated the difference of the area ratio of three types of MCs, and the correlation between the area ratio of MCs region and the HU value of endplate. We compared different Modic types in the axial plane of the endplate and in the sagittal plane of the vertebral, and found that the area ratio of MCs region showed no statistical difference (*p* > 0.05). We used Pearson correlation analysis, and found that the HU value was not significantly associated to the area ratio of MCs region in MCs type I and III (*p* > 0.05). However in MCs type II, there is a significantly positive correlation between these two parameters (*p* < 0.01). The area ratio of MCs region was positive correlated between the axial plane of the endplate and the sagittal plane of the vertebral (*p* < 0.001).

**Table 4:** The area ratio of Modic change region

Type of Mcs	MCs type I	MCs type II	MCs type III
Mean area ratio of endplate	32.91% ± 14.23%	43.46% ± 19.00%	35.26% ± 21.19%
Mean area ratio of sagittal	17.75% ± 9.40%	23.16% ± 11.02%	24.89% ± 14.35%
Mean HU value of endplate	543.70 ± 130.02	561.90 ± 158.40	729.87 ± 125.83

### Evaluation of degree of disc degeneration

In our study, we found that all intervertebral discs adjacent to the MCs had different degrees of degeneration. According to Pfirrmann classification of disc degeneration, we found that there were 2 (1.3%) discs with grade II degeneration, 23 (14.6%) discs with grade III,83 (52.5%) with grade IV and 50 (31.6%) with grade V in T2-weight MRI images. Degree of degeneration was not significantly different in different Modic types (*p* > 0.05). But we found a significant correlation between the degree of degeneration and the area ratios of MCs region (*p* < 0.001), and a significant correlation between the degree of degeneration and the HU value of endplate (*p* < 0.05). In the area ratios of MCs region, we found a significantly statistical difference between grade V degeneration and grade III/ IV (*p* < 0.001), and no statistical difference between grade III and IV (*p* > 0.05). Larger area ratio of MCs region was associated with more severe the degeneration. We observed no statistical difference between the HU value of endplate with grade III, IV and V degeneration (*p* > 0.05), although the average HU value increased with the grades of degeneration. Grade II degeneration was not compared due to a small sample size. Detailed data were shown in Table 5.

**Table 5:** Evaluation of degree of disc degeneration

Degree of degeneration	Grade II	Grade III	Grade IV	Grade V
Disc number	2	23	83	50
area ratio of endplate	13.89% ± 2.14%	32.23% ± 15.28%	36.93% ± 16.19%	54.24% ± 18.23%**
area ratio of sagittal	5.60% ± 2.15%	19.66% ± 9.68%	20.44% ± 11.24%	28.14% ± 9.65%**
HU value of endplate	440.30 ± 42.14	544.74 ± 157.17	560.54 ± 161.91	609.58 ± 151.46

**A significantly statistical difference between grade V degeneration and grade III/ IV (*p* < 0.001)

## Discussion

In clinical practice, chronic lower back pain (LBP) is a common disease closely related to the degeneration of lumbar spine. Many pathological factors of lumbar spine, such as intervertebral disc degeneration and its surrounding soft tissue diseases, inflammatory diseases, spinal nerves pathological changes, can cause lower back pain. MCs are pathological changes of the vertebral endplate and bone marrow, which can be diagnosed on MRI scans, and are considered clinically relevant because of their association with chronic LBP [8–10]. MCs are divided into three types, and the most common is type II MCs. Type III MCs were thought to be associated with endplate sclerosis. Type II MCs were regarded as fat deposition since their initial description. Type I MCs were thought to indicate inflammatory reactions [2, 3]. It is intuitive that the HU values of type III MCs regions will be higher than that of normal endplate. However, few studies have revealed the changes of HU values in type I and type II MCs.

In our study, all types of MCs were included to evaluate the change of HU values in different types of MCs. Our findings show that the HU values of MCs regions were significantly higher than those of the corresponding regions in adjacent vertebra without-MCs, regardless of the endplate region and bone marrow region. The HU value in type III was higher than that in type I and II. In type I and type II, we found HU values slight decreased in very few cases. As we all know, higher HU value is associated with more severe endplate sclerosis. Our results showed that osteosclerosis may coexist with fat deposition and inflammatory reactions. In addition, the consistency between axial plane and sagittal plane suggested the error of our measurement was negligible. Our study was a quantitative measurement and provided accurate information. We could infer that the pathological process of MCs might be osteosclerosis. Kuismaet al, Liu et al. and Xu et al. found that endplate sclerosis existed in all types of MCs [11–13]. Their research has the same conclusion as ours. We consider that three types of MCs are different stages of the same pathological process. MCs I and MCs II can transform to each other to some extent, and it is possible for them to gradually change to MCs III, which means that the endplate and bone marrow have severe osteosclerosis [14–16]. MCs III is the most stable state. Of course, MCs in most patients may not transform to type III in the end, but rather stop at MCs type I or II. We believe that MCs I and II are the dynamic stages, where the HU value, various inflammatory factors, fatty and bone marrow metabolism and other pathological processes are in a dynamic changes, while MCs III represents a steady state.

In the past, Dual-energy X-ray absorptiometry (DXA) is the gold standard for bone mineral density quantification [17, 18]. Recently, many studies reported a strong positive correlation between HU value and bone mineral density. Researchers were able to estimate bone mineral density using diagnostic CT images and showed that HU value is more sensitive sometimes than DXA in the assessment of lumbar vertebral osteoporosis [19–21]. Our study show that HU value increased with T-score, too. According to the previous studies and our results, we believe that the higher the HU value, the greater the bone strength. It is obvious that the bone strength of the endplate and vertebral body directly affects the support of the cage in lumbar fusion operation. If we can carefully measure the HU value of the endplate and vertebral body before the lumbar fusion operation, we would predict the subsidence risk of the cage after the operation. If the endplate and the vertebral body is strong enough, we can use a simpler and minimally invasive operation, such as oblique lumbar interbody fusion (OLIF) stand-alone without the posterior fixation assisted, to solve the serious lumbar problem.

As we know, area ratios are regularly used in pathology as indexes of lesion severity, such as in estimating degree of myocardial infarctions, cerebral infarction, burn injury, and idiopathic femoral head osteonecrosis. Likewise, many studies have reported a positive correlation between MCs area ratios and lower back pain, especially MCs type I and type II [22, 23]. Our findings show that the area ratios of MCs region had a significant correlation with the degrees of disc degeneration. As the area ratio becomes larger, the disc degeneration becomes more severe. Without doubt, the degree of degeneration determines the degree of chronic lower back pain. We think that as the area ratio of MCs region gets larger, chronic lower back pain will become more severe. We also found that there was a positive correlation between the area ratio of the endplate and the area ratio of the sagittal plane, which proved that the diffusion of MCs was simultaneous to the endplate and the vertebral body. In our study, we also found that HU value of endplate was significantly associated with the area ratio of MCs region in type II, but not in MCs type I and III. The reason behind this phenomenon is unknown and may require further research.

There were some limitations to our study. First, this is a single-centre, retrospective study. Our sample size is not large enough. In particular, there are not enough cases of MCs type I and type III. Secondly, our study focused on the phenotypic analysis of endplate changes on MRI and CT scans and we did not study the related pathological process and mechanical analysis. Thirdly, errors exist in HU value measurement because of subjective measurement. Fourthly, our samples are all in-patients with severe pain beyond endurance, who need surgical intervention, which lead to a relatively high pain score. For this reason, analyzing the correlation between HU value, MCs area and pain remains difficult. Further study may needed in the future.

## Conclusions

The HU values of the vertebral endplate and bone marrow increased in endplates with all type of MCs. Besides in type III, osteosclerosis may exist in type I and II MCs.HU value of endplate had a significantly positive correlation with BMD of lumbar spine. Higher area ratio of MCs region is correlated with more severe intervertebral disc degeneration.

## Data Availability

The datasets used and/or analysed during the current study available from the corresponding author on reasonable request.

## Abbreviations

MCs: Modic changes
BMD: Bone mineral density
DXA: Dual-energy x-ray absorptiometry
BMI: Body mass index
LBP: Lower back pain
OLIF: Oblique lumbar interbody fusion
